# Detection, referral and control of diabetes and hypertension in the rural Eastern Cape Province of South Africa by community health outreach workers in the rural primary healthcare project: Health in Every Hut

**DOI:** 10.4102/phcfm.v10i1.1610

**Published:** 2018-04-11

**Authors:** Angela A. Morris-Paxton, Paul Rheeder, Rose-Marie G. Ewing, Dillon Ewing

**Affiliations:** 1 Donald Woods Foundation, Mbashe, South Africa; 2 Nelson Mandela Metropolitan University, South Africa; 3 Department of Internal Medicine, Faculty of Health Sciences, University of Pretoria, South Africa; 4 Donald Woods Foundation, London, United Kingdom

## Abstract

**Background:**

Non-communicable diseases, mainly cardiovascular diseases, diabetes, cancer and chronic respiratory diseases, are responsible for approximately 63% of all deaths occurring worldwide in any given year. The majority of these deaths have occurred in low- and middle-income countries (LMICs). The latest World Health Organization (WHO) report shows that the increase in diabetes is also most pronounced in the LMICs. The South African Labour and Development Research Unit estimated a 9% prevalence within the adult population in 2016. In the Eastern Cape Province, hypertensive heart disease has become the second most common cause of death, followed by diabetes, the third most common cause of death.

**Aim and setting:**

The aim of this study was to report on the follow-up of patients in the community with known hypertension or diabetes or who were deemed at-risk (as identified during a prior community-wide survey).

**Methods:**

Data were collected via a household primary health screening, monitoring and follow-up process, which included taking anthropometric measurements, blood pressure (BP) and blood glucose and referring to clinics for further testing and treatment where necessary.

**Results:**

Of the 1885 participants followed up by the community health outreach workers, 1702 were known to be hypertensive and 183 were deemed at-risk [of these, only 24 (13.2%) had normal or high normal systolic BP readings]. There were 341 participants with diabetes and 34 at-risk of diabetes [of these, 28 (82%) had levels of 11 mmol/l or higher at follow-up]. There was a significant improvement in BP and glucose control over repeated visits.

**Conclusion:**

In this rural area of the Eastern Cape, South Africa, the follow-up of patients with hypertension or diabetes as well as those individuals at-risk adds value to hypertension and glucose control.

## Introduction

Of the 56 million deaths that occurred globally during 2012, 68% (38 million) was because of non-communicable diseases (NCDs), principally cardiovascular diseases, diabetes, cancer and chronic respiratory diseases.^1^ Nearly 80% of these NCD deaths (29 million) occurred in low- and middle-income countries (LMICs).^1^ The latest World Health Organization (WHO) report on diabetes shows a doubling in the prevalence of diabetes between 1980 and 2014. The increase in diabetes prevalence is most pronounced in the LMICs. The percentage of deaths attributed to high blood glucose in those aged 20–69 years in LMICs was 60.5% in men and 45.6% in women.^2^ The International Diabetes Federation (IDF) estimated that there were 2.28 million people living with diabetes in South Africa in 2015 (7% of adults).^3^ Shen et al. evaluated pre-diabetes and diabetes in certain areas in South America, South Asia and South Africa and found that the prevalence of diabetes and pre-diabetes was 14.0% and 17.8% in the Southern Cone of Latin America, 9.8% and 17.1% in Peru, 19.0% and 24.0% in South Asia and 13.8% and 9.9% in South Africa.^4^

Mills et al. evaluated studies of blood pressure (BP) in 90 countries and found that in 2010, 31.1% (95% confidence interval [CI] 30.0%–32.2%) of the world’s adults had hypertension, 28.5% (27.3%–29.7%) in high-income countries and 31.5% (30.2%–32.9%) in LMICs. An estimated 1.39 (1.34–1.44) billion people had hypertension in 2010: 349 (337–361) million in high-income countries and 1.04 (0.99–1.09) billion in LMICs. From 2000 to 2010, the age-standardised prevalence of hypertension decreased by 2.6% in high-income countries, but increased by 7.7% in LMICs. During the same period, the proportions of awareness (58.2% vs. 67.0%), treatment (44.5% vs. 55.6%) and control (17.9% vs. 28.4%) increased substantially in high-income countries, whereas awareness (32.3% vs. 37.9%) and treatment (24.9% vs. 29.0%) increased less, and control (8.4% vs. 7.7%) even slightly decreased in LMICs.^5^

A worldwide goal for the prevention and control of NCDs has been proposed to complement the previously set Millennium Development Goals,^6^ with the global voluntary target for 2020 set at a 25% relative reduction in overall mortality from cardiovascular diseases, cancer, diabetes or chronic respiratory diseases, with the accompanying target of an additional 2% per year reduction in death rates attributed to the main chronic diseases (heart disease, stroke, cancer, diabetes and chronic respiratory diseases).^6,7^

### Non-communicable diseases in Africa

In sub-Saharan Africa, the burden of NCDs could exceed that of infectious diseases in the coming decades.^8^ Even allowing for the uncertainties surrounding HIV, the relative rise of the importance of preventing NCDs is set to increase.^8^ Exacerbating the situation is the increase in westernised lifestyle patterns such as tobacco and alcohol consumption alongside the reduction of physical activity and the changes in dietary intake.^8^ Nutrition-related non-communicable diseases (NRNCDs) are rising across the board in developing countries, including western and sub-Saharan Africa, largely because of the transition in nutritional intake to a more westernised diet.^9^ Studies have shown that the prevalence of NRNCDs is highest among the poor, with urbanisation positively linked to obesity; however, insulin resistance and dyslipidaemia were found to be spread equally among both rural and urban dwelling communities.^9^ In both Benin and Burkina Faso, the dual problem of undernutrition and diseases of excess creates a double burden for the health care systems.^9^

### Non-communicable diseases in South Africa

Despite being the economic powerhouse of the continent, the NCD disease burden in South Africa is similar to the rest of the sub-Saharan region. The Study of Global Ageing (SAGE) reported that, in South Africa, close to 70% (> 8 million people) of people 45 years and older were hypertensive, with treatment coverage at 27.5%.^10^ As quoted by Mayosi,^11^ some data are available for rural South Africa. Evidence from the Agincourt study emphasises the emergence of NCDs, including differences by sex in rural South Africa.^12^ Mortality related to these diseases was well established by the early 1990s, with age-standardised death rates per 100 000 population of 209 in men and 172 in women for 1992–1994.^11^ Examination of the leading causes of death in men and women aged 50–64 years, and 65 years and older, draws attention to the rise in deaths from vascular disease, notably in women aged 65 years and older.^13^

Aligned to the global goal, the South African Strategic Plan for the Prevention and Control of NCDs aims to reduce by at least 25% the relatively premature mortality (< 60 years) from NCDs by 2020.^14^ Maher et al. discuss priorities for developing countries in the global response to NCDs, in which they contrast the individual versus the public health approach (the latter being of great importance in South Africa).^15^ The individual approach relies on a patient consulting a primary care provider.^15^ In many areas of the country, particularly rural areas, primary health care is not always accessible to those requiring it, and therefore a public health approach would be far more effective. This approach has three key elements: identifying and addressing modifiable risk factors; screening for common NCDs; and diagnosing, treating, following up and, when necessary, referring patients with common NCDs, using standard protocols.^15^

In 2010, Honourable Minister Dr Aaron Motsoaledi, the Minister of Health of South Africa, introduced the ‘Re-engineering of Primary Health Care’ model that would be implemented through health promoting, proactive household and community-focused interventions.^16^ Practically, this meant the formation of municipal ward outreach teams, district specialist teams and school health teams, an initiative that provides an opportunity for the implementation of a public health approach for controlling NCDs.^16^

### Non-communicable diseases in the Eastern Cape

Regarding the burden of NCDs in the Eastern Cape, Day et al. reported that in the metro of Nelson Mandela Bay the age-standardised mortality rate (deaths per 100 000 population) for hypertensive heart disease was 129.8 (second leading cause of death) and for diabetes it was 45.5 (eighth leading cause) with Buffalo City at 118.3 (third leading cause) for hypertensive heart disease and 44.9 (12th leading cause) for diabetes.^17^ Since 1994 the South African government implemented the Wild Coast Spatial Development Initiative (SDI) in the rural coastal region that is under investigation in this study.^18^ Notwithstanding some improvements to access to water and sanitation, the area still falls well below both national and regional standards for access to clean and potable water, employment and health care services.^18^ The poverty level and lack of development serve to further increase the health inequality that already exists, and those with lower incomes were found to visit the government health services less often because of distance and cost of transport.^18^

The Donald Woods Foundation (DWF), based on the decentralised ward-based primary health care strategy, embarked on the Health in Every Hut (HiEH) programme in 2012, with financial support from the Lilly NCD Partnership. A service level agreement between the Eastern Cape Department of Health (ECDoH) and the HiEH was implemented in seven clinic catchment areas in the Mbashe sub-district of the Amathole District (Eastern Cape Province) and three clinic catchment areas of the King Sabata Dalindyebo sub-district of the OR Tambo District (Eastern Cape Province), South Africa.^19^ The aim of this programme was to improve the health status of inhabitants, which included early detection, referral and treatment of people found to be at high risk of hypertension and diabetes, or having high readings at screening.

## Research methods and design

### Study design

The study reports on the findings of cross-sectional, descriptive data collected after the initial household screening survey,^20^ the clinic visits and the follow-up of patients. The period of screening relevant to the study was conducted by a dedicated team of community health outreach workers (CHOWs) from June 2013 to August 2016. This study will report on the hypertension and diabetes outcomes in terms of detection, referral, treatment and response.

#### Study setting, participant selection and sampling procedure

The development and implementation of the HiEH programme by the DWF has been reported elsewhere, prior to this report.^20^ The study was conducted using anonymous data gathered from seven clinic catchment areas of the Mbashe sub-district of the Amathole District and three clinic catchment areas of the King Sabata Dalindyebo sub-district of the OR Tambo District (Eastern Cape Province), South Africa.

In essence, this entailed the recruitment and training of CHOWs for 13 weeks to do home visits in order to collect appropriate demographic and health data, and measure weight, height, BP and capillary glucose. Prior to 2015, glucose measurements were only conducted in those individuals deemed at high risk, for example, body mass index (BMI) > 25. Thereafter, random blood glucose (RBG) measurements were routinely conducted on all people aged 16 years and over. If participants had a BP value greater than 159/99 mm Hg, or a glucose value greater than 11 mmol/l, they were referred to their local clinic to confirm diagnosis and commence treatment as soon as possible. If the values were lower but still abnormal (for BP 140/90–158/98 mm Hg and for glucose 8–10.9 mmol/l), participants were flagged as being at-risk, and were followed up with repeated measurements by the CHOWs on a weekly basis. At the nearest clinic, referred participants were seen and retested. If diagnoses were confirmed, then treatment was initiated and information provided to the HiEH team for home follow-up by the CHOWs. Follow-up visits entailed re-checking the patients’ BP, capillary glucose, ascertaining if those patients who had been referred to the clinic, had made the clinic visit, had collected their medication and were taking the medication appropriately.

### Data collection

Screening, referrals and follow-up of participants were the responsibility of selected and trained CHOWs, 24 screening CHOWs, and in the case of clinical referral and follow-up, a team of 80 community-embedded CHOWs. Screening and embedded CHOWs were recruited exclusively from the communities in which they resided. Individual applications were submitted to clinics, following a broad-brush advocacy campaign across the relevant clinic catchment areas. In the case of each catchment area, 80–100 applicants were shortlisted for assessment.

Once the assessment process, conducted over 2–3 days, was completed, the recruitment panel selected 65–80 applicants to attend the first 2 weeks of training, during which time they continued to be assessed against predetermined criteria. At the end of the first 2 weeks, 65 applicants were taken forward to complete the 14-week training course, and the assessment continued. At the end of 14 weeks, depending on the need of the catchment area, 24 applicants were selected to be either screening or embedded CHOWs for each catchment area.

The training that was provided matched the training provided by the DOH for their community health workers (CHWs) and the CHOWs also received extensive training on Computer Literacy, Lay Counselling Skills and First Aid. Once trained, CHOWs were placed in the field and were supervised by CHOW team leaders on the first level and CHOW supervisors the second level. Each catchment area had an initial 24 CHOWs for screening and then an eventual 10–12 embedded CHOWs, a supervisor and a team leader. Screening CHOWs were deployed to visit people in their homes for health screening – bringing primary health care to the homesteads of the local population. Screening covered seven different areas: hypertension, diabetes mellitus, maternal and child health, HIV, tuberculosis (TB), dementia and identification of orphaned and vulnerable children. Any persons with abnormal readings were referred to an embedded CHOW for a re-screening or to a clinic for treatment. Embedded CHOWs were deployed in their own communities to do regular follow-up (usually weekly) on those people who had high readings (glucose and BP) or who had been referred from screening to the clinics.

#### The screening survey

Data were collected during screening, patients’ clinic visits and embedded CHOWs’ follow-up visits. Screening included the collection of biographical information, physical environment, health history, maternal and child health, HIV, TB and dementia. In addition, NCD-related data – obesity, diabetes and hypertension – were collected, which are the subject of the study. Clients were provided with a written referral to the health facility if necessary.

#### Anthropometric measurements, blood pressure and blood glucose

Weight was measured with a Safeway bathroom scale (without shoes, jackets and coats) and height with a tape measure. Prior to June 2015, capillary random glucose was measured with Accu-Chek Active on all clients with a BMI of 25 or more; thereafter, On Call EZ glucometers were utilised. Blood pressure measurement was conducted with clients sitting on a chair with arm at the level of the heart, after at least 5 min rest, with either a Rossmax device wrist measurement (during 2013–2015) or a Tensoval device using the upper arm (2015 to date).

### Data collection instruments

Data collection instruments were developed for screening, clinic recording and follow-up visits, viz., screening data were captured directly by screening CHOWs onto a Libre Office database, which was synchronised with the main DWF server daily. This continued from June 2013 to September 2015, after which the data set was amended and data from September 2015 to June 2016 were captured onto MS Excel. Clinic data were captured onto MS Excel, at clinics, by DWF data capturers and synchronised with the DWF server on a weekly basis. Follow-up data were largely collected on paper and delivered daily to the DWF monitoring and evaluation team for capturing.

### Data verification

Verification of the data was done at the DWF centre, which included checking for missing records, anomalies and outliers. In addition, data were triangulated with written records: duplicate CHOW referral forms were checked against the electronic data for completeness. Google Earth maps were consulted to check that all households in an area had been visited by screening CHOWs. Meetings were held with groups of embedded CHOWs to check electronic follow-up data against written records that CHOWs had kept as backup. Clinic data were checked by a dedicated member of the clinic support team, who checked electronic records against the patients’ clinic files. Verified data for the full period of June 2013 to August 2016 were linked and imported into a single database.

### Data analysis

Of the 17 350 adult patients initially screened for hypertension, for whom data were available and analysed, 1885 patients were followed up by CHOWs between June 2014 and August 2016. For the sake of NCD evaluation, the analysis was restricted to people aged 16 years or older, and adults who were HIV positive, pregnant or had active TB were excluded from the analysis. Diabetes and hypertension were defined if a person reported the condition, or if they were on medication for the condition at the time of questioning. Data were analysed using Statistica and SPSS V 20. Summaries of descriptive statistics and paired group comparisons are provided, which were made using the paired Student’s *t*-test for continuous data and the McNemar’s test for paired proportions.

Blood pressure categories were classified using the South African Hypertension guidelines.^21^ Glucose categories were classified using cut-off points recommended by the Society for Endocrinology, Metabolism and Diabetes of South Africa (SEMDSA) guideline^22^ for diabetes testing and not the cut-off points used for screening. This was done in order to make comparisons with other studies possible. Age categories were in accordance with the South African National Health and Nutrition Examination Survey (SANHANES-1).^23^ Body mass index categories were done according to WHO definitions.^24^ Blood pressure categories were classified using the South African Hypertension guidelines.^21^

### Ethical considerations

The programme and evaluation was approved by the University of Pretoria, Faculty of Health Sciences Research Ethics Committee: The monitoring and evaluation of the Donald Woods Foundation’s Health in Every Hut programme (341/2016).

## Results

### Hypertension

At the first follow-up visit, 1702 patients (90.29%) had known hypertension and were receiving medication, and 183 patients (9.71%) were at-risk, but not receiving medication at the first visit. The categories used for the assessment of BP risk were according to the South African Guidelines for Hypertension,^21^ which give the cut-off points for both systolic and diastolic BP values. On analysis, it was found that many patients had a diastolic blood pressure (DBP) lower than the cut-off point for hypertension, but a higher than cut-off point systolic blood pressure (SBP). For example, 416 patients who were receiving medication had a DBP below 84 mm Hg (normal SBP 120–129 mm Hg DBP 80–84 mm Hg), and an SBP above 129 mm Hg (high normal SBP 130–139 mm Hg DBP 85–89 mm Hg). Using the DBP as the deciding factor would have placed these patients in the normal category, whereas they were high normal or hypertensive, and may have been at-risk for a higher category of hypertension. As the aim was to monitor at-risk patients, the higher of the two readings was utilised (in this instance, the SBP), the cut-off marker for designation of hypertension categories, for the purposes of monitoring and repeated follow-up visits. At the first follow-up visit, the following results were found.

In monitoring the diagnosed hypertensive patients, it was found at the first follow-up visit that 44.42% were controlled hypertensives, falling into the normal or high normal category, whilst a further 37.6% were mildly hypertensive (at low risk) and 17.98% were moderate to high risk uncontrolled hypertensives (Table 1).

**TABLE 1:** Systolic blood pressure categories at first follow-up visit – Hypertensive patients.

SBP first visit (mm Hg)	Category	Frequency	Percentage (%)	Cumulative (%)
< 129	Normal	457	26.85	26.85
130–139	High normal	299	17.57	44.42
140–159	Hypertension stage 1	640	37.60	82.02
160–179	Hypertension stage 2	246	14.45	96.47
> 180 (180–220)	Hypertension stage 3	60	3.53	100.00

**Total**	**-**	**1702**	**100.00**	**100.00**

SBP, systolic blood pressure.

With respect to patients at-risk and undiagnosed at the first follow-up visit, 13.11% (24 patients) were normal or high normal and not at significant risk; however, 38.8% fell into the category of being mildly hypertensive, and a further 48.09% were moderate to high risk uncontrolled hypertensives (Table 2).

**TABLE 2:** Systolic blood pressure categories at first follow-up visit – At-risk patients.

SBP (mm Hg)	Category	Frequency	Percentage (%)	Cumulative (%)
< 129	Normal	9	4.92	4.92
130–139	High normal	15	8.19	13.11
140–159	Hypertension stage 1	71	38.80	51.91
160–179	Hypertension stage 2	59	32.24	84.15
< 180 (180–220)	Hypertension stage 3	29	15.85	100.00

**Total**	**-**	**183**	**100.00**	**100.00**

SBP, systolic blood pressure.

All patients received between 1 and 31 follow-up visits (median 5 visits, range 1–31); 739 patients had been referred to the clinic at the first follow-up visit, of whom 555 (75%) had confirmed clinic visits. Follow-up visits had an approximate 20–30-min duration and were conducted on a weekly basis. These visits involved re-checking BP and medication regimens and that instructions for taking medication were followed correctly as prescribed. As many follow-up visits were given as was necessary for the individual patient to be stable and be able to self-manage their condition.

At the final follow-up visit, all patients were receiving medication (including those who had initially tested normal or low risk). Of the 1728 patients who had received two or more visits, 23 were re-referred to the clinic by the CHOW and 223 patients had confirmed clinic visits, of which 200 had self-referred.

A comparison of the data between the first and final follow-up visits shows that, at the final follow-up visit (Table 3), a larger number of patients tested fell into the normal, high normal and hypertension stage 1 categories, and fewer patients fell into the hypertension stage 2 or 3 categories, compared to the first follow-up visit.

**TABLE 3:** Systolic blood pressure categories at final follow-up visit.

Systolic pressure (mm Hg)	Category	Frequency	Percentage (%)	Cumulative (%)
< 129	Normal	477	25.31	25.31
130–139	High normal	318	16.87	42.18
140–159	Hypertension stage 1	837	44.40	86.58
160–179	Hypertension stage 2	208	11.03	97.61
180–220	Hypertension stage 3	45	2.39	100.00

**Total**	**-**	**1885**	**100**	**100.00**

Comparing the first visit mean SBP of 143.7 mm Hg and the final visit mean SBP of 141.0 mm Hg showed a difference of 2.7 mm Hg (*p* < 0.001). The mean number of visits was 5.9 (*N* = 1885). Excluding the patients who were normal at baseline from both groups (diagnosed and at-risk), of the remaining 1419 patients who were followed up by the CHOWs, an exact McNemar’s test comparing BP categories at first and last visits determined that there was a significant positive difference in lowered BP between the pre- and post-CHOW follow-up visits (*p* < 0.001*)*.

As patients became stable in their BP measurements over the 2-year period, the number of visits was reduced, and for those whose BP remained high, the CHOW visits continued. There was a strong positive correlation between the systolic BP and the number of CHOW follow-up visits (Spearman’s rho, *r* = 0.282, *p* < 0.001*).* Patients who continued to have a high BP were medicated and monitored until their BP normalised.

### Diabetes

At the first follow-up visit, 341 known diabetic patients were receiving medication and 34 patients who were not on medication were deemed to be at high risk, with their diabetic status unknown. Postprandial RBG was taken by the CHOWs at each of the follow-up visits. Glucose categories were classified using cut points recommended by the SEMDSA diabetes guideline^22^ for diabetes testing in order to make comparisons with other studies possible. Whilst it is appreciated that these are not the same as the cut-off points used for diagnosis and treatment, it was not practical in the field to take fasting glucose and 2-h postprandial glucose measurements. The purpose of the CHOW visits was that of monitoring those at-risk, and maintenance of treatment regimes in previously diagnosed patients. Patients with unusually high or unstable RBG readings were referred to the nearest fixed-point Department of Health (DOH) clinic for further investigation. At the first follow-up visit, 140 (41%) of the diabetic patients had stable blood glucose levels, whilst the remaining 201 (59%) had high blood glucose levels (Table 4).

**TABLE 4:** Blood glucose measurements at first follow-up visit – Diabetic patients.

Random blood glucose (mmol/l)	Frequency	Percentage (%)	Cumulative (%)
< 7.7	48	14.08	14.08
7.7–11	92	26.98	41.06
> 11 (11.1–31)	201	58.94	100.00

**Total**	**341**	**100.00**	**100.00**

Of the patients deemed at-risk at first visit (Table 5), only six (17.56%) had stable RBG levels, whilst 28 (82.35%) had high RBG levels.

**TABLE 5:** Blood glucose measurements at first follow-up visit – At-risk patients.

Random blood glucose (mmol/l)	Frequency	Percentage (%)	Cumulative (%)
< 7.7	2	5.88	5.88
7.7–11	4	11.77	17.65
> 11 (11.1–31)	28	82.35	100.00

**Total**	**34**	**100.00**	**100.00**

All patients received between 1 and 34 follow-up visits (median 6 visits, range 1–34 visits); 186 patients had been referred to the clinic at the first follow-up visit, of which 152 had confirmed clinic visits. As for the hypertensive patients, all follow-up visits to the patients with diabetes were between 20 and 30 min duration and involved re-checking capillary glucose, medication regimens and instructions for taking medication. Health workers additionally checked that dietary advice given at the clinic was being followed. At the final follow-up visit, 357 patients were receiving medication and for 18 patients medication was deemed unnecessary. Of the 337 patients who had received two or more visits, three were re-referred to the clinic by the CHOW; however, 44 patients had confirmed clinic visits of whom 41 had self-referred. A comparison of the data between the first and final follow-up visits shows that at the final follow-up visit (Table 6), a larger number of patients tested fell into the ranges ≤ 7.7 mmol/l (normal) and 7.8–11 mmol/l (moderately high), and fewer patients fell into the > 11 mmol/l (high) category, compared to the first follow-up visit (McNemar’s test, *p* < 0.001).

**TABLE 6:** Blood glucose measurements at final follow-up visit.

Blood glucose (mmol/l)	Frequency	Percentage (%)	Cumulative (%)
< 7.7	66	17.60	17.60
7.7–11	123	32.80	50.40
> 11 (11.1–31)	186	49.60	100.00

**Total**	**375**	**100.00**	**100.00**

Comparing the first visit mean RBG of 13.51 mmol/l and the final visit mean RBG of 12.35 mmol/l demonstrated a mean difference of 1.16 mmol/l (*p* < 0.001). Excluding the participants who were normal at baseline, the remaining 325 patients who were diabetic or at-risk were followed up by the CHOWs.

As patients became stable in their blood glucose measurements, the number of visits was reduced, and for those whose blood glucose remained high, the CHOW visits continued weekly. A strong positive correlation was found between the blood glucose readings and the number of CHOW follow-up visits (Spearman’s rho, *r* = 0.291, *p* = < 0.001). Patients who continued to have high blood glucose readings were medicated and monitored until their blood glucose readings normalised

## Discussion

The goal of antihypertensive therapy is the treatment and control of BP, without compromising quality of life.^25^ Hypertension is notoriously asymptomatic and often under-diagnosed, with frequent adverse outcomes for patients.^25^ The prevalence of type 2 diabetes in South Africa is currently estimated at 9%; however, about half of the cases are estimated to be undiagnosed and untreated and this can result in an increase in complications in the future, including blindness and amputations, if left unchecked.^26^ Reducing the burden of NCDs has been a neglected area of universal health coverage, and the maldistribution of health care workers between urban and rural health services is a global concern.^25^ Any shortage of health care workers in a given area, especially the rural areas, is a barrier to the implementation of universal health care coverage, one of the most basic human rights.^27^ This leads to the overloading of services in urban areas, when people migrate for health-related reasons, with adverse consequences for both patients and practitioners.^27^

The data collected by the CHOWs in the HiEH programme provide a unique snapshot of the challenge of NCDs in the rural Eastern Cape. The re-engineering of health care model, with the focus on teams visiting homes in villages, provides valuable data on the health status in the communities in which they serve. This study was conducted in a very challenging environment, necessitating health care workers to travel long distances over inaccessible terrain to reach participants in their homes. The research participants were either hypertensive, diabetic or at-risk for hypertension or diabetes.

The results of this study demonstrate that with time and regular CHOW visits to patients in their homes, monitoring of both those who have been diagnosed with hypertension and diabetes and those at-risk for disease pays off. Those at-risk have been placed on medication when necessary, and those receiving medication have become better controlled, with a decreased chance of health complications. Task sharing has been widely used internationally for the control of diseases such as HIV and TB and is emerging as an effective strategy in the control of cardiovascular diseases.^28^ A study conducted in Argentina found that a CHW-led intervention to enable low-income adults with uncontrolled hypertension to regain control and manage their BP was successful.^28^ In South Africa, an assessment of a CHW pilot programme to improve the management and control of hypertension and diabetes in Emfuleni, Gauteng, had similar results, in that the control of hypertension and hypertension or diabetes co-morbidity was shown to have improved by CHW home visits.^29^ For diabetic patients with no co-morbidity, however, there appeared to be marginally better control for those attending the local clinic than those visited at home.^29^

In South Africa, effective HIV and TB care in rural and peri-urban areas has been improved with the implementation of CHW home visits.^29^ The Pilani mother–mentor system of CHWs visiting homes in Cape Town found success in improving both HIV-related health and nutrition outcomes, although this is limited mainly to maternal health and does not embrace wider aspects of primary health care.^30^ A similar CHW home-visit programme has been instituted, in an area adjacent to the one in the current study (King Sabatha Dalindyebo district) and, whilst there are no official evaluation outcomes, the rural hospital instituting the system has been praised for its improvement of community care and outreach services.^30^ A qualitative evaluation of child-health-focused CHW visits in the rural Eastern Cape of South Africa also found that there was significant improvement in integration with formal health care facilities and social services to provide for vulnerable children.^31^

Countries may differ in their means to integrate and coordinate government and civil society agencies, but the net result is the same, that is, an expanded primary health care capacity.^32^ In the context of changing requirements, from less urgent infectious disorders to the monitoring and treatment of non-communicable chronic disease, cooperative engagement is a vital means of delivering health and social welfare services.^32^

### Limitations

This study had its limitations. One problem is the difficulty in obtaining comparative clinic visit data. The shortage of health care personnel impacts not only those directly involved in health care provision, but also those who support the provision in an administrative capacity. In this respect, the writers acknowledge the fact that comparative clinic visit data, and follow-up of patients who visited the DOH clinics, may have added depth and quality to this study, but was not possible to obtain. The second limitation is that this study was conducted in a limited geographical area of 1000 km^2^ and thus the results cannot be generalised to the rural population in the country as a whole.

### Recommendations

It is clear from this study that CHOWs have the potential to make a positive difference in the long-term health and wellness of a deep rural community. In light of the reforms intended by the DOH re-engineering of primary health care delivery, the authors would recommend that this type of home-based, health monitoring and support should be considered for inclusion in the regional and national strategy for health service delivery.

## Conclusion

The study demonstrates that a small but significant positive difference has been made to the control of BP and blood glucose between the first and final CHOW visits. The most important finding in the study was that the CHOW follow-up visits to patients are an independent factor in maintaining or improving BP and blood glucose control in both those diagnosed with hypertension or diabetes and those at-risk. Particular attention should be paid to those individuals with continued high risk determined by either very high glucose or BP readings as they would be most prone to complications.
