# Erratum: Detection, referral and control of diabetes and hypertension in the rural Eastern Cape Province of South Africa by community health outreach workers in the rural primary health care project: Health in Every Hut

**DOI:** 10.4102/phcfm.v10i1.1828

**Published:** 2018-11-29

**Authors:** Angela A. Morris-Paxton, Paul Rheeder, Rose-Marie G. Ewing, Dillon Woods

**Affiliations:** 1 Donald Woods Foundation, Mbashe, South Africa; 2 Nelson Mandela Metropolitan University, South Africa; 3 Department of Internal Medicine, Faculty of Health Sciences, University of Pretoria, South Africa; 4 Donald Woods Foundation, London, United Kingdom

In the author list of this article initially published, Dillon Woods surname was unintentionally misprinted as ‘Ewing’. The correct surname is ‘Woods’. The publisher sincerely regrets this error and apologises for any inconvenience caused. 

